# Evaluation of Five Tests for Sensitivity to Functional Deficits following Cervical or Thoracic Dorsal Column Transection in the Rat

**DOI:** 10.1371/journal.pone.0150141

**Published:** 2016-03-02

**Authors:** Nitish D. Fagoe, Callan L. Attwell, Ruben Eggers, Lizz Tuinenbreijer, Dorette Kouwenhoven, Joost Verhaagen, Matthew R. J. Mason

**Affiliations:** 1 Laboratory for Neuroregeneration, Netherlands Institute for Neuroscience, an Institute of the Royal Academy of Arts and Sciences, Amsterdam, The Netherlands; 2 Center for Neurogenomics and Cognitive Research, Neuroscience Campus Amsterdam, VU University, Amsterdam, The Netherlands; Virginia Commonwealth University, UNITED STATES

## Abstract

The dorsal column lesion model of spinal cord injury targets sensory fibres which originate from the dorsal root ganglia and ascend in the dorsal funiculus. It has the advantages that fibres can be specifically traced from the sciatic nerve, verifiably complete lesions can be performed of the labelled fibres, and it can be used to study sprouting in the central nervous system from the conditioning lesion effect. However, functional deficits from this type of lesion are mild, making assessment of experimental treatment-induced functional recovery difficult. Here, five functional tests were compared for their sensitivity to functional deficits, and hence their suitability to reliably measure recovery of function after dorsal column injury. We assessed the tape removal test, the rope crossing test, CatWalk gait analysis, and the horizontal ladder, and introduce a new test, the inclined rolling ladder. Animals with dorsal column injuries at C4 or T7 level were compared to sham-operated animals for a duration of eight weeks. As well as comparing groups at individual timepoints we also compared the longitudinal data over the whole time course with linear mixed models (LMMs), and for tests where steps are scored as success/error, using generalized LMMs for binomial data. Although, generally, function recovered to sham levels within 2–6 weeks, in most tests we were able to detect significant deficits with whole time-course comparisons. On the horizontal ladder deficits were detected until 5–6 weeks. With the new inclined rolling ladder functional deficits were somewhat more consistent over the testing period and appeared to last for 6–7 weeks. Of the CatWalk parameters base of support was sensitive to cervical and thoracic lesions while hind-paw print-width was affected by cervical lesion only. The inclined rolling ladder test in combination with the horizontal ladder and the CatWalk may prove useful to monitor functional recovery after experimental treatment in this lesion model.

## Introduction

Lesions of the dorsal funiculus of the spinal cord sever the ascending sensory fibres that originate in the dorsal root ganglia and terminate in the brainstem, and also damage the dorsal component of the motor corticospinal tract. Targeting the ascending dorsal columns (DC) is useful because transganglionic labelling from the sciatic nerve allows sprouting or regeneration to be assessed in a specific labelled subpopulation of fibres, for which lesion completeness can be confirmed in the brainstem. Because it targets the fibres of primary sensory neurons, it is a useful model to study the potential for neuron-intrinsic factors to promote axon regeneration. These fibres can be induced to sprout beyond the distal lesion border by a peripheral nerve lesion (the conditioning lesion effect) [1–5]. The model is also attractive because gene expression in the primary sensory neurons can be manipulated (e.g. by the use of adeno-associated viral vectors [6–8]).

While this model is well suited for studying axon sprouting or regeneration in the CNS anatomically, it is also useful in a spinal cord injury model to be able to quantify the effects on functional deficits resulting from an experimental intervention. Suitable functional tests would ideally show a detectable and sustained deficit in lesioned animals compared to sham-lesioned animals, so that functional improvements resulting from experimental treatments may be detectable. The use of functional tests with transection lesions of the DC has until now been fairly limited, probably because a transection lesion of the DC results in mild functional deficits that are transient and difficult to detect. Tests developed for nociception typically stimulate paw withdrawal via local spinal cord circuitry which is left intact by a dorsal column lesion. The sensory afferents ascending in the DC carry information to the brainstem comprising tactile information, discriminatory touch, vibration, and proprioception [9]. Useful tests for the ascending dorsal column pathway therefore should comprise tasks expected to be sensitive to these sensory functionalities.

Functional deficits of the forepaws were detected after a crush lesion of the DC [10] at C4, with an increase in foot-slips seen on a beam walk test, a grid walk test and footprint analysis performed with ink and paper. However, following a dorsal column transection lesion only transient deficits of the fore paws were found, and no deficit in hind paw function [11]. In a later study these authors again found deficits of the fore paws and only small and transient deficits in hind paw function after this type of injury [12].

We here compare five functional tests for use in this model using a deep medial lesion of the dorsal column performed with microscissors. This has the advantage that it reliably severs the L4-L5 sensory component of the dorsal column while sparing the lateral white matter tracts. We selected four previously described tests for comparison that have previously proved useful for the detection of functional improvement in spinal cord injury models. These were the adhesive tape removal test, horizontal ladder, rope crossing test and CatWalk gait analysis [13–17]. The sticky tape removal test assumes that a lesioned animal will have impaired fine touch sensation and take longer to notice and remove a small piece of tape placed on the rear paw. The other tests are based on the idea that a decline in proprioception and possibly subtle motor deficits resulting from a dorsal column lesion will result in less coordinated stepping on the horizontal ladder or rope, and an altered gait on an even surface.

A dorsal column lesion also impairs discrimination of texture [18,19]. Based on this, a reaching task was described which exploits the deficit in fine touch and discrimination of texture [19]. This test involves the animal reaching for and being required to discriminate between a food item and a similarly shaped but roughly textured non-food item, a testing paradigm which requires food restriction and is limited to the forepaws. We designed a new test, named the inclined rolling ladder, where optimal performance should depend on tactile discrimination and proprioceptive feedback simultaneously. This consists of ladder rungs on a 45 degrees angle that have an immobile heavily textured half and a smooth, freely rolling half. We hypothesised that after training, animals with intact sensation of texture would be able to discriminate between rough-fixed and smooth-rolling bars, and either choose the textured sections or adapt their stepping action when they detected a smooth (and thus rolling) step. This may result in a detectable increase in slip rate in lesioned animals. Compounding the loss of texture discrimination would be the loss of proprioception resulting in more errors in lesioned animals. We reasoned that such a test might be more sensitive than existing tests to the loss of sensory information carried up the spinal cord. To determine the optimal lesion level to produce functional deficit, animals received a C4 or T7 complete bilateral dorsal column transection or a sham laminectomy at the same level and underwent the described tests for a duration of 8 weeks. Both lesion locations will disrupt the transmission along the dorsal column of sensory information from the hindlimbs, while a lesion at C4 would be expected to block signals from the thoracic regions and partially from the forelimb [20].

Furthermore, we have applied current statistical techniques to analyse the datasets generated. Functional testing data are longitudinal and many tests yield essentially binary outcome measures (e.g. slips or successful steps). This type of data is often analyzed with t-tests or ANOVA at individual timepoints, or with repeated measures ANOVA for longitudinal analysis. However such data may violate some assumptions of these techniques, for example that the data are sampled from populations that are normally distributed, and that each group is sampled from a population with the same variance (homoscedasticity). In particular dichotomous categorical outcome data, such as counts of slips versus successful steps, is problematic because the data are expected to have a binomial distribution. In addition repeated measures ANOVA cannot be used with unbalanced data (i.e. differing group sizes) or deal with missing values. This does not combine well with functional testing in spinal cord injury experiments where subjects may have to be removed early. However whole-time course comparisons can be more sensitive to deficits than comparing individual timepoints. We use here the general linear model extensions the linear mixed model (LMM) for comparing longitudinal data, and binomial generalized LMM (GLMM) which is suitable for longitudinal binary outcome data [21,22], to test whether functional outcome data differ between lesioned and sham-lesioned animals.

## Materials and Methods

### Experimental animals and surgical procedures

All experimental procedures and postoperative care were carried out with approval from the animal experimentation ethical committee of the Royal Netherlands Academy of Sciences. A total of 24 female Fischer rats (9–12 weeks old; Harlan, Horst, the Netherlands) were used. Animals were housed under standard conditions with food and water ad libitum, and a 12-hour: 12-hour light:dark cycle. Animals received either a lesion at C4 (n = 6), a sham lesion at C4 (n = 6), a lesion at T7 (n = 6), or a sham lesion at T7 (n = 6). Animals were anesthetized using isoflurane. Following an incision along the dorsal midline, a laminectomy at C4 or T7 was performed to expose the spinal cord and the dura mater was opened. To minimize compression damage of the spinal cord we first inserted a 30G needle at 1mm (at C4) or 0.6mm (at T7) lateral to the midline on either side to a depth of 1.6mm (at C4) or 1.4mm (at T7). The resulting hole was then enlarged by inserting a 27G needle to the same depth. Finally the tips of a pair of microscissors were inserted in the same holes to the same depth and then closed. Sham-operated animals received only the laminectomy. The muscles overlying the spinal cord were loosely sutured together with a 5–0 suture and the wound closed. Animals were allowed to recover at 37°C and received postoperative analgesia (Temgesic 0.03 ml/100 g body weight s.c.; Schering-Plough, Maarssen, the Netherlands), and to survive for eight weeks after injury.

Three days before perfusion, the animals were re-anesthetized and the left sciatic nerve was exposed. Animals were injected with 3μl cholera toxin subunit B (CTB; 10mg/ml) (103B, List Laboratories Inc., Campbell, CA) in the sciatic nerve to transganglionically label ascending dorsal column axons in the spinal cord. After 3 days animals were injected with a lethal dose of pentobarbital and transcardially perfused with 0.9% saline followed by 4% paraformaldehyde (PFA) in phosphate buffer. Brain stems and spinal cords were post-fixed in 4% PFA for 3–4 hours at room temperature and transferred to 30% sucrose in phosphate-buffered saline, and were frozen in Tissue-Tek OCT (4583; Sakura Finetek Holland) the following day.

### Immunohistochemistry

Brain stems were sectioned transversely at 50μm on a cryostat and free floating sections were blocked with 2% horse serum with 0.1% triton X100, and incubated for 72 hours with goat anti-CTB (1:80.000; 703, List Laboratories Inc., Campbell, CA), followed by biotinylated horse anti-goat (1:300; Vector Labs), ABC reagent (1:200; Vector Labs), with washes in 1xTBS between each step, and finally developed using diaminobenzidine with nickel ammonium sulphate. Sections were then mounted onto Superfrost Plus slides (Menzel-Gläser), dehydrated and embedded with Entellan. Spinal cords were sectioned parasagittally at 20μm on a cryostat onto Superfrost Plus slides (Menzel-Gläser), and stained with the same procedure.

### Functional testing

All functional tests (described below) were performed before injury to get a baseline measurement and two or three days and seven days after injury, followed by weekly measurements. Four experimenters carried out the tests, working in pairs, and each test was carried out consistently by the same experimenters throughout the time course. The different tests were all performed in the same order each week and at the same approximate time of day. Animals had a daily pre-training period of 2 weeks prior to baseline measurements and all had mastered accurately traversing the respective platform/ rope/ ladder and were comfortable being handled for the tape removal test. Testers were blinded with regard to which animals were lesioned or sham-lesioned. In the rope, horizontal ladder and inclined rolling ladder, any indication of mis-stepping including 'stutter steps' was counted as a slip.

### CatWalk gait analysis

The CatWalk XT gait analysis system is a platform locomotor test in which animals cross a 7 cm wide walkway with a glass floor (131 cm long) located in a darkened room [16,17]. The glass floor is illuminated from the side, so the light is scattered where a paw touches the glass. Three runs per animal were recorded by a high-speed camera (100 frames/s) that captures the paw prints from below. All four paws were automatically labelled using the Catwalk software and were checked afterwards by a blinded experimenter for gait analysis. For each animal the base of support, stride length, swing time, print width, mean pixel intensity and maximum contact area for the hind paws were measured using the CatWalk software package. These parameters were chosen based on previous literature [10] and because they could be expected to partially depend on proprioceptive function. Mean values of left and right hind paws were taken. All values were normalized to baseline measurements for each animal for plotting.

### Rope-crossing test

The rope-crossing test was carried out as previously described [15]. A 1.25-meter long rope with a diameter of 4 cm was set up between two platforms. The animal was required to traverse the rope three times and the number of slips and steps with the left hind paw were live-scored by two blinded observers. Counts of slips and successful steps were used for statistical analysis. For plotting of the data, the number of slips was divided by the total number of steps for each run and averaged over three runs to calculate the mean error ratio.

### Inclined rolling ladder test

**Fig 1:**
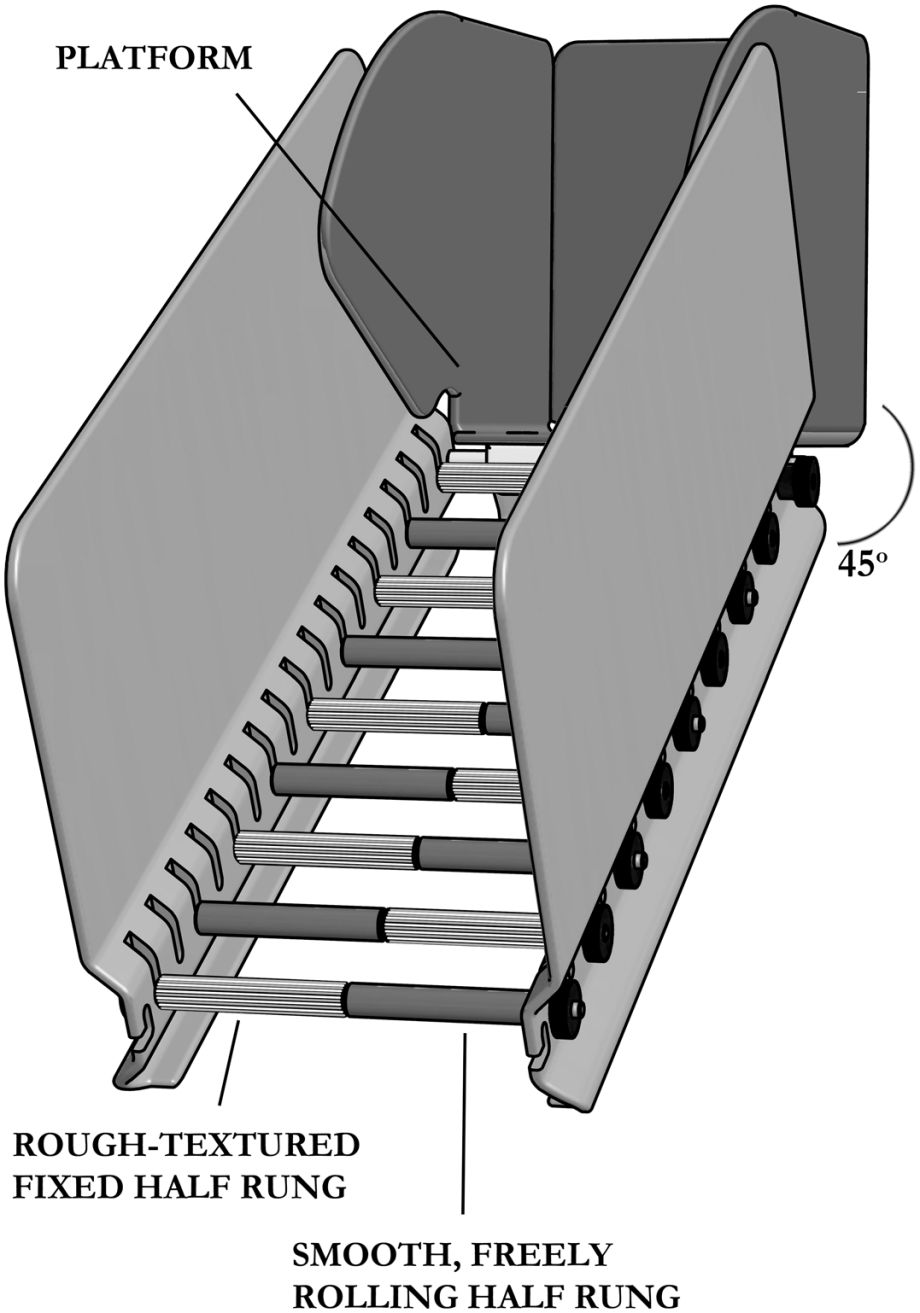
Schematic diagram of the inclined rolling ladder. The inclined rolling ladder consists of a ladder constructed at a 45 degrees angle with rungs that have an immobile, heavily textured half and a smooth, freely rolling half. Animals are required to walk up the ladder to the platform. After training, animals with intact sensation of texture should be able to discriminate between rough, fixed and smooth, rolling rungs, and either choose the textured sections or adapt their stepping action when they detected a smooth (and thus rolling) rung.

The inclined rolling ladder is a 34 cm long ladder set at a 45 degree angle with 9 adjustable rungs with half smooth (rolling) and half rough (and fixed) rungs (see Fig 1), intended to simultaneously test tactile sense and proprioception. Animals are required to walk up the ladder to the platform. We hypothesized that after dorsal column injury animals cannot discriminate between the smooth rolling rungs and the fixed, textured rungs, leading to an increase in errors. In order to prevent a learning effect the orientations of the ladder rungs were randomized at each time point. Three runs per animal were video recorded and analysed by two independent blinded observers. Successful steps, slips and the type of rung from which these occurred (rough or smooth) were scored.

Two related measures were used: the Good Smooth Steps measure, defined as successful steps on smooth bars vs. any other step type (slips from smooth bars or any steps from rough bars), and the Slips measure, defined as slips vs. successful steps on smooth bars only. Counts of successful/failed steps per run by these definitions were used for statistical analysis.

For plotting of the data, to calculate the mean ratio for the Good Smooth Steps measure the number of successful steps on smooth rungs was divided by the total number of steps for each run and averaged over three runs. To calculate the mean error ratio for the Slips measure, the number of slips was divided by the total number of steps on smooth rungs for each run and averaged for three runs.

### Horizontal ladder test

The horizontal ladder, adapted from the gridwalk test [14] is a 0.9 meter long horizontal ladder with a diameter of 15.5 cm. The rungs of the ladder are adjustable with a possible gap of 3.5–5.0cm and were randomly adjusted for each time point to prevent a learning effect. Two independent observers live-scored left and right rear slips and total left rear steps which was multiplied by two to determine total steps. Counts of slips and successful steps were used for statistical analysis, while for plotting the total number of slips was divided over the total number of steps for each run and averaged for three runs to calculate the mean error ratio.

### Tape removal test

Sensory function was tested using the tape removal test [13]. Animals were held with both hind-paws exended and a piece of tape (Kip Hochkrepp, #803, Bocholt, Germany) of 15 x 15 millimetres was affixed to the palm of the left hind paw. The time until the animal had detected the tape was measured in three individual trials to calculate the mean sensing time. Trials in which the animals did not detect the tape were stopped after 180 seconds and this was taken as the time recorded.

### Statistical analysis

The rope test, the horizontal ladder test and the inclined rolling ladder were all analysed as follows. Because we scored individual steps as either a successful step or a slip, the data follows a binomial distribution. The whole time-courses of lesioned and sham groups were compared using binomial generalized linear mixed models (GLMM) [22], with lesioned/sham ('LESION'; set to true in lesioned animals post-surgery, false otherwise) and operated/unoperated ('OPERATED'; set to false before surgery, true after surgery) as fixed factors, animal as a random factor and time as a continuous covariate.

Binomial GLMMs were fitted in R using package *lme4* [23,24] with the *glmer* function. The model formulae used were of the following form:

outcome ~ LESION * time + OPERATED + (time|animal) (1)

outcome ~ LESION+ time + OPERATED + (time|animal) (2)

outcome ~ LESION+ time + OPERATED + (1|animal) (3)

outcome ~ time + OPERATED + (1|animal) (4)

The bracketed terms in these models indicate the 'random effects' and account for the presence of repeated measurements in the estimation of the effect size and significance of INT and LESION. 'Outcome' for binomial GLMMs is a two-column list of counts of successes/fails per run as described in the individual models above.

We refer to INT as the interaction term of LESION with time (in Model 1). We calculated p-values for INT, which represents differences in the evolution of outcomes over time, and LESION, the unconditional main effect of lesioning.

Significance of specific parameters in LMMs and GLMMs can be determined by comparing a model containing the parameter of interest to a reduced model without it [21]. Thus INT was assessed by comparing Model 1 and Model 2, while LESION was assessed by comparing Model 3 and Model 4.

LESION represents the overall effect of lesioning on the outcome, while INT represents the difference in slope of the outcome between the two groups, i.e. in this case a difference in the speed of recovery.

Accurate calculation of p-values for GLMMs is not straightforward and currently the most accurate method is by model comparison using parametric bootstrap [22,25]. p-values were calculated in this way for INT and LESION against the null hypothesis that each parameter is zero, using R package *pbkrtest* [26]. Between 1000 and 20000 simulations were used.

In the rope (at T7) and horizontal ladder tests an observation-level random effect was included to account for overdispersion (i.e. greater variance in the data than expected for a binomial distribution) [27, 28]. Significance of differences at individual time-points were calculated in a factorial 2x2 binomial GLMM treating baseline and the specific time point only. The significance of the interaction of group (lesioned or sham-lesioned animals) with timepoint was calculated.

For the CatWalk parameters and the tape removal test, the time-courses of lesioned versus sham were compared using linear mixed models (LMMs) with the *lme4* function *lmer*. Model formulae were as above. Standard regression diagnostics (quantile plots of the residuals vs the normal distribution, plots of residuals vs fitted values) were carried out for the data fitted with linear mixed models.

P-values for the INT and LESION parameters of the LMMs were calculated by model comparison using package *pbkrtest* [26], with the Kenward-Roger method. For the tape test we used the log of the withdrawal time as the data were expected to follow an exponential distribution. Significance of differences at individual time-points were calculated similarly to above using a LMM.

### Power analysis

Power analysis was performed to determine the power of each test to detect differences at the α = 0.0.5 level, with n = 6 animals per group, for LESION, by data simulation (see [29]). For each test outcome, 1000 simulated datasets were generated using the *simulate* function of *lme4*, using parameters extracted from the fitted model (model 2 above or equivalent) above using the *getME* function of *lme4*. For GLMMs total step counts were generated randomly for each simulation, from a normal distribution with a mean and standard deviation taken from the experimental data. The simulations were then analyzed as for the collected data, and the proportion of datasets that were found significantly different (p<0.05) was calculated. For GLMM analysis, approximate p-values using the Likelihood Ratio test were used rather than parametric bootstrap, as the latter would be unfeasible, following the example of [30].

## Results

### Lesion completeness

**Fig 2:**
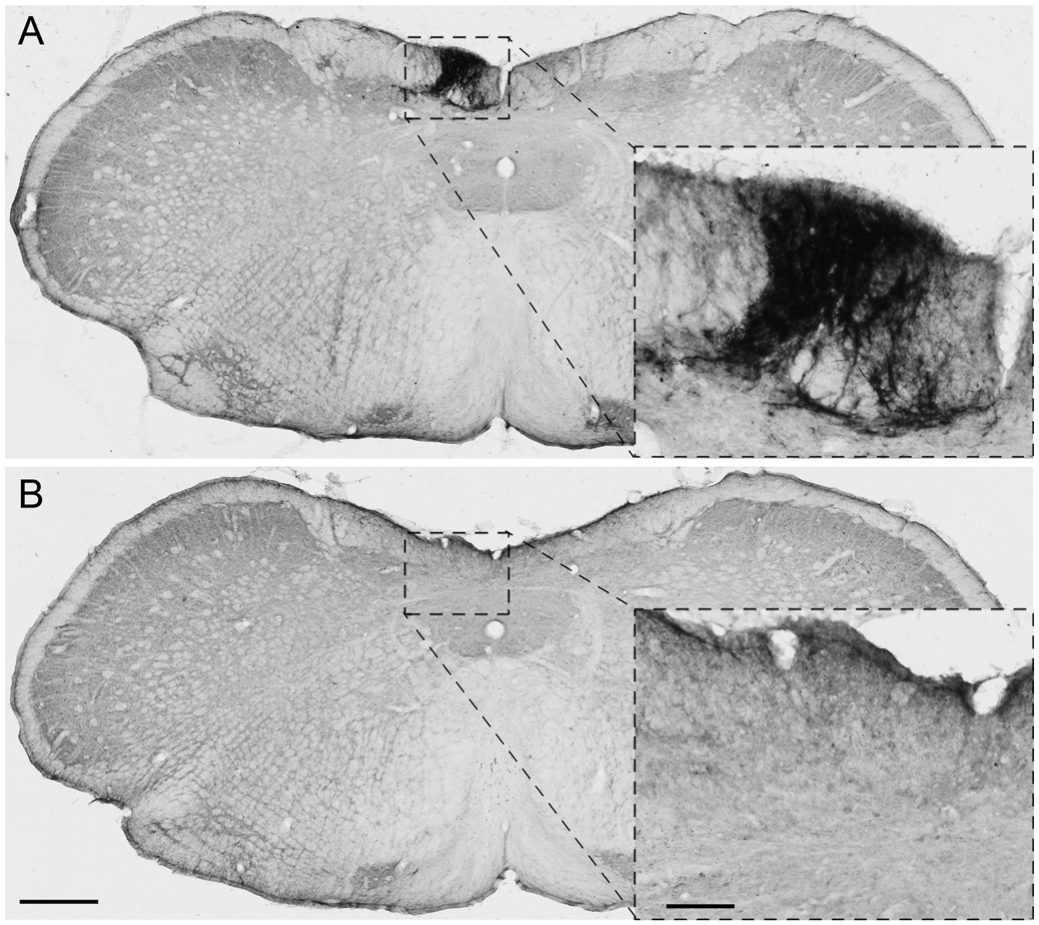
Histological evaluation of lesion completeness. Animals received a dorsal column transection at T7 or C4 level or sham surgery and survived eight weeks after which ascending sensory axons were transganglionically labelled with cholera toxin-B (CTB). Sections were processed for immunohistochemistry for CTB. **(A)** Section of the brain stem of an animal that received a sham lesion. The higher magnification image shows that many CTB-positive axons are visible in the nucleus gracilis of the brain stem. **(B)** Section of the brain stem of a lesioned animal. Bar = 500μm. The higher magnification image shows that no CTB-positive fibres were detected in the brain stem. Bar = 50μm.

**Fig 3:**
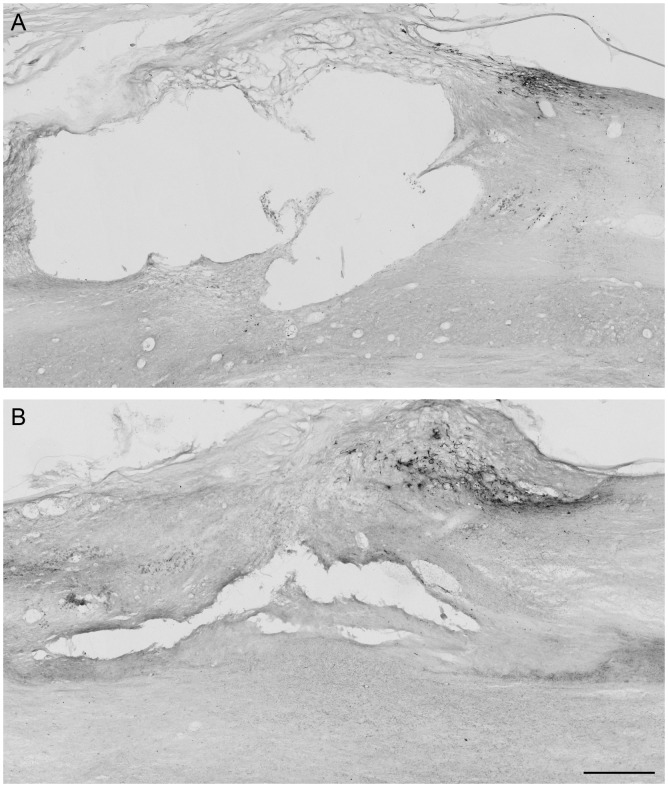
Histological evaluation of the spinal cord after injury. Animals received a dorsal column transection or a sham lesion at T7 or C4 level and survived eight weeks after which sensory axons were transganglionically labelled with CTB. Sections were processed for immunohistochemistry for CTB. **(A)** Parasagittal section of the lesion center in the spinal cord from an animal that received a cervical dorsal column injury. **(B)** Section of spinal cord from an animal that received a thoracic dorsal column injury. Bar = 100μm.

Animals received a dorsal column transection at T7 or C4 level or sham surgery. To check for lesion completeness, sensory afferents were transganglionically traced from the left sciatic nerve and the dorsal column nuclei in the brain stem checked for spared fibres by histological analysis. We observed CTB-labelling in the brain stem of one animal in the group that received a thoracic lesion, indicating that sparing of axons occurred. This animal was excluded from further analysis. As expected, positive staining in the brain stem was observed in all sham animals (Fig 2). In spinal cord sections we observed CTB-positive axons up to, but not crossing the lesion site in animals that received a cervical and thoracic dorsal column transection (Fig 3).

### Tape removal test

**Fig 4:**
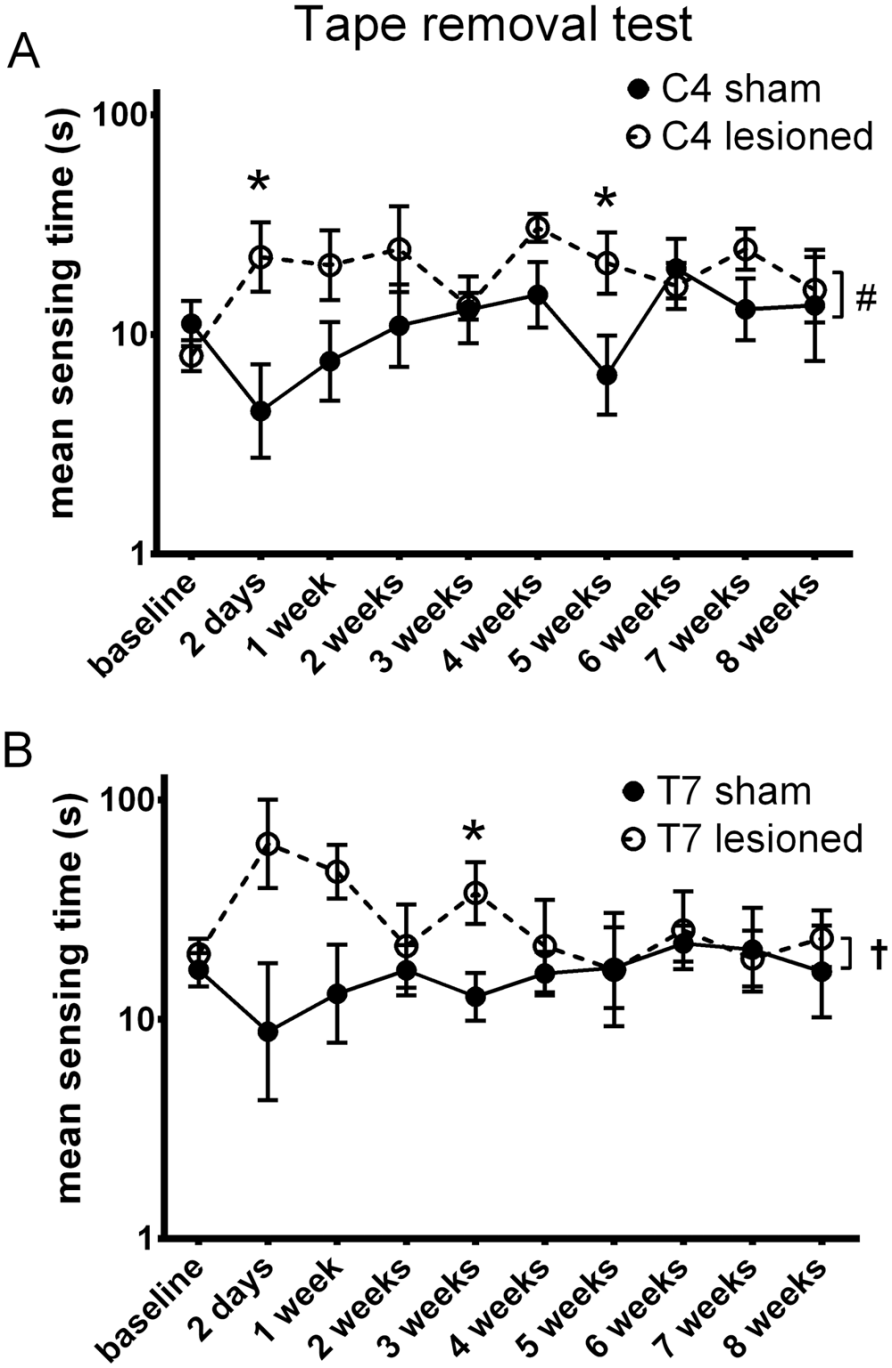
Tape removal test. The tape removal test was performed in order to evaluate sensory dysfunction of the left hind paw up to eight weeks after cervical and thoracic dorsal column injury. Graphs show the mean time to tape removal on a logarithmic scale. We observed overall deficits in the ability to sense the adhesive tape on the left hind paw in both **(A)** C4 and **(B)** T7 injured animals compared to the sham groups. Whole time -course comparisons: # p < 0.05 for LESION † p < 0.05 for INT. *p<0.05 for single time-point comparisons. Error bars are SEM.

The tape removal task tests touch perception from the left hind paw. The time until the animal notices a piece of adhesive tape placed on the palm of the left hind paw was measured by a blinded observer. We observed an overall significant increase in the time it takes to sense and find the adhesive tape for both the C4 and T7 injured animals (linear mixed model, p = 0.01 for C4 LESION; C4 INT was n.s.; p = 0.04 for T7 INT, T7 LESION was n.s.) compared to the sham groups over the whole time period of 8 weeks (Fig 4), but at individual time points differences were significant at only 2 days and 5 weeks (C4) and 3 weeks (T7). Parameter p-values, F statistics and degrees of freedom are summarized in Table 1.

**Table 1:** Summary of F statistics, degrees of freedom and parameter significance, calculated by the Kenward-Roger approach, in LMMs used to analyse CatWalk outcomes and the tape removal test.

Test	Outcome	Lesion level	Parameter	numerator DF	denominator DF	F statistic	p-value
Catwalk	Base of support	C4	LESION	1	22	14.3	0.001
			INT	1	33.8	17.4	0.0002
		T7	LESION	1	76	7.03	0.01
			INT	1	9.8	0.73	n.s.
	Print width	C4	LESION	1	13.3	24.12	2.70E-04
			INT	1	18.5	3.3	n.s.
		T7	LESION	1	38.3	0.38	n.s.
			INT	1	11.3	0.95	n.s.
	Swing time	C4	LESION	1	35.9	7.11	0.011
			INT	1	13.8	0.04	n.s.
		T7	LESION	1	20	2.45	n.s.
			INT	1	67.2	1.46	
	Stride length	C4	LESION	1	28.1	0.87	n.s.
			INT	1	21.8	3.1	n.s.
		T7	LESION	1	11.7	3.65	n.s.
			INT	1	26.6	0.008	n.s.
	Mean pixel intensity	C4	LESION	1	13.5	0.24	n.s.
			INT	1	19.7	0.6	n.s.
		T7	LESION	1	19.7	2.54	n.s.
			INT	1	13.3	1.6	n.s.
	Maximum contact area	C4	LESION	1	14.2	2.41	n.s.
			INT	1	38	4	n.s.
		T7	LESION	1	16.3	8.71	0.009
			INT	1	17.2	1.1	n.s.
Tape		C4	LESION	1	25.9	7.31	0.012
			INT	1	17.9	4.02	n.s.
		T7	LESION	1	27.3	3.12	n.s.
			INT	1	19.1	4.81	0.04

### Rope crossing test

**Fig 5:**
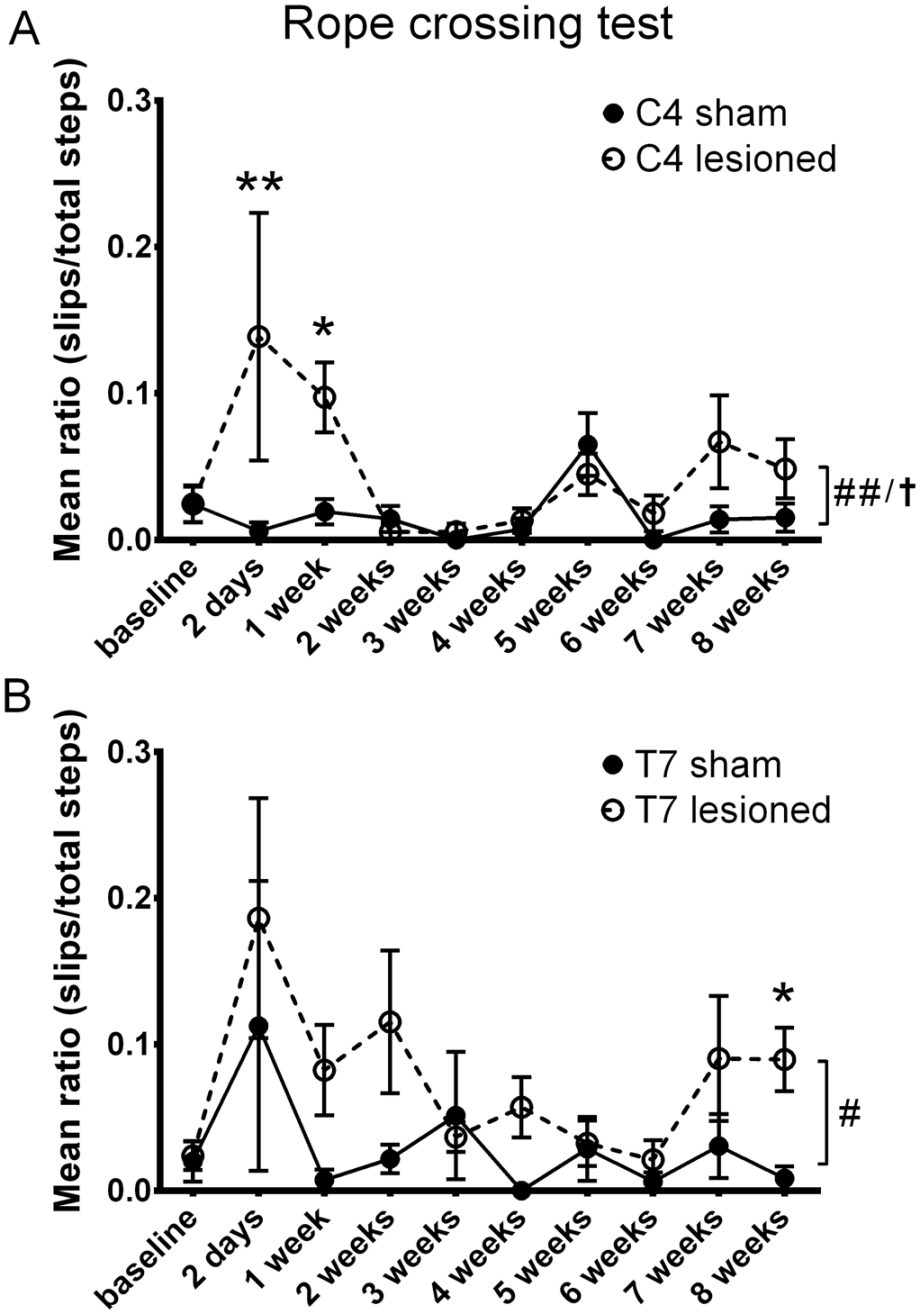
Rope crossing test. Sensorimotor dysfunction was assessed using the rope crossing test for eight weeks after cervical and thoracic dorsal column injury. Graphs show the mean ratios of errors to total steps taken. In animals that received either a **(A)** C4 or T7 **(B)** DC injury we observed an overall significant increase in the error rate compared to the sham groups. Whole time -course comparisons: # p < 0.05, ## p < 0.01 LESION † p < 0.05 for INT. *p < 0.05, ** p < 0.01 for single time-point comparisons. Error bars are SEM.

In this test the rats were allowed to cross a rope between two platforms. The number of steps taken and the number of errors made with the left hind paw were counted by two blinded observers. Over the time course in both the cervical and thoracic injury groups there was a significant increase in the error rates (binomial generalized linear mixed model, p = 0.03 for C4 INT, p = 0.004 for C4 LESION; p = 0.03 for T7 LESION, T7 INT was n.s.) compared to the sham animals (Fig 5). At individual time-points cervically injured animals showed a significant deficit at 2 days and 1 week only. Parameter p-values are summarized in Table 2.

**Table 2:** Summary of parameter significance, calculated by parametric bootstrap, in binomial GLMMs used to analyse horizontal ladder, inclined rolling ladder and rope tests.

Test	Outcome	Lesion level	Parameter	p-value
Horizontal ladder		C4	LESION	4.0E-04
			INT	0.0015
		T7	LESION	0.025
			INT	n.s.
Inclined ladder	Good smooth steps	C4	LESION	0.002
			INT	n.s.
	Slips	C4	LESION	0.01
			INT	n.s.
	Good smooth steps	T7	LESION	0.002
			INT	n.s.
	Slips	T7	LESION	0.001
			INT	n.s.
Rope		C4	LESION	0.004
			INT	0.028
		T7	LESION	0.030
			INT	n.s.

### Horizontal ladder test

**Fig 6:**
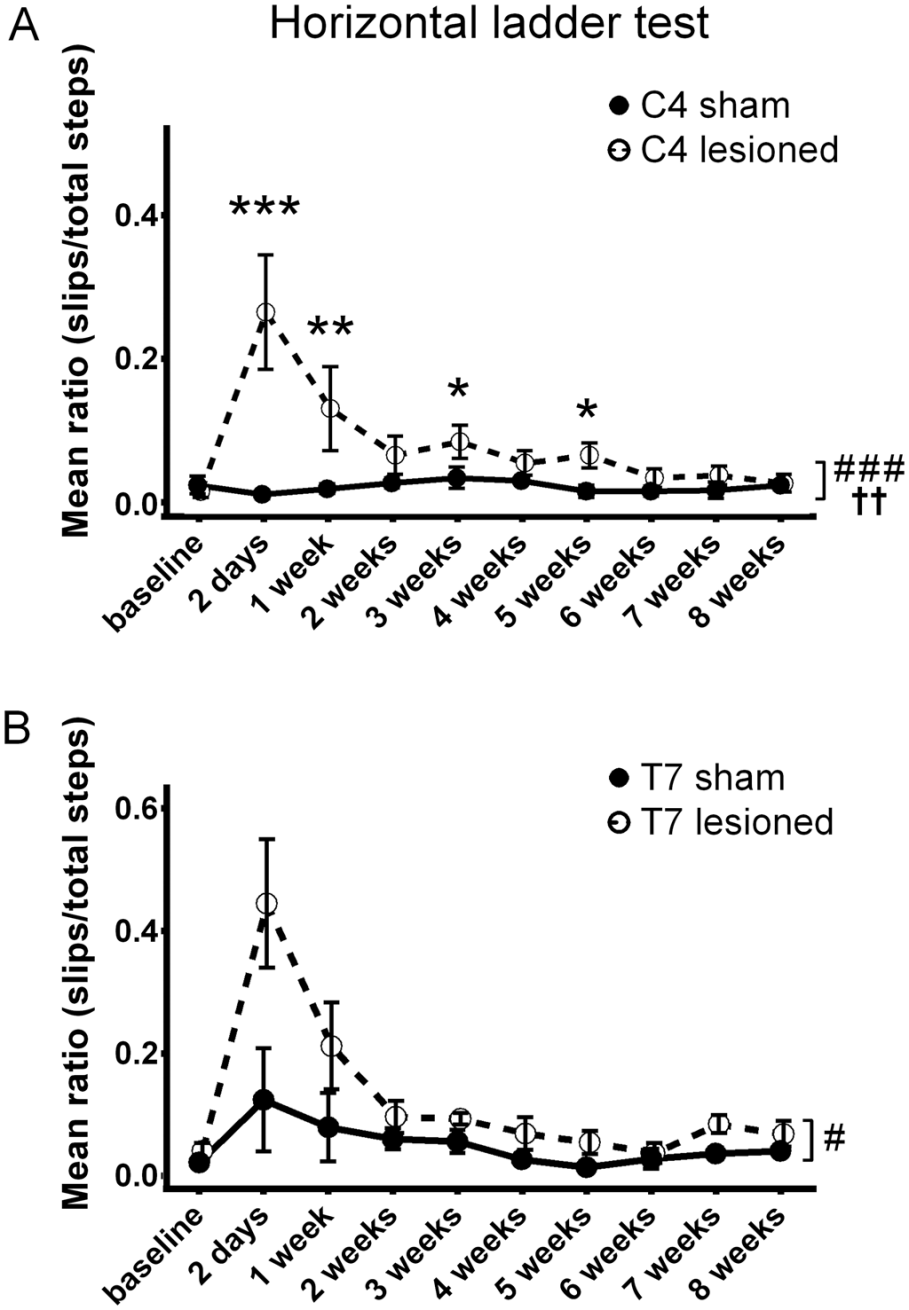
Horizontal ladder test. Animals were allowed to cross a horizontal grid with gaps of randomized size to assess dysfunction during eight weeks after cervical or thoracic dorsal column lesion. Graphs show the mean ratios of errors to total steps taken. For both **(A)** C4 and **(B)** T7 lesioned animals we observed an overall increase in the error rates compared to sham controls. Whole time -course comparisons: # p < 0.05, ### p < 0.001 for LESION; †† p < 0.01 for INT.*p<0.05, **p<0.01, ***p<0.001 for single time-point comparison. Error bars are SEM.

Animals were allowed to cross a horizontal ladder with rung gaps of randomized length to assess dysfunction. The number of steps and errors made with the left hind paw was scored by two blinded observers up to eight weeks after injury. Over the time course in both the cervical and thoracic injury groups there was a significant increase in the error rates compared to the controls (Fig 6), although the effect size was larger for the cervical group (see 'Comparing Tests' below) (binomial generalized linear mixed model, p = 0.0017 for C4 INT, p = 0.00035 for C4 LESION; T7 INT was n.s., p = 0.025 for T7 LESION. At individual time-points cervically injured animals showed a significant deficit at 2 days, 1 week, 3 weeks and 5 weeks. Parameter p-values are summarized in Table 2.

### CatWalk gait analysis

**Fig 7:**
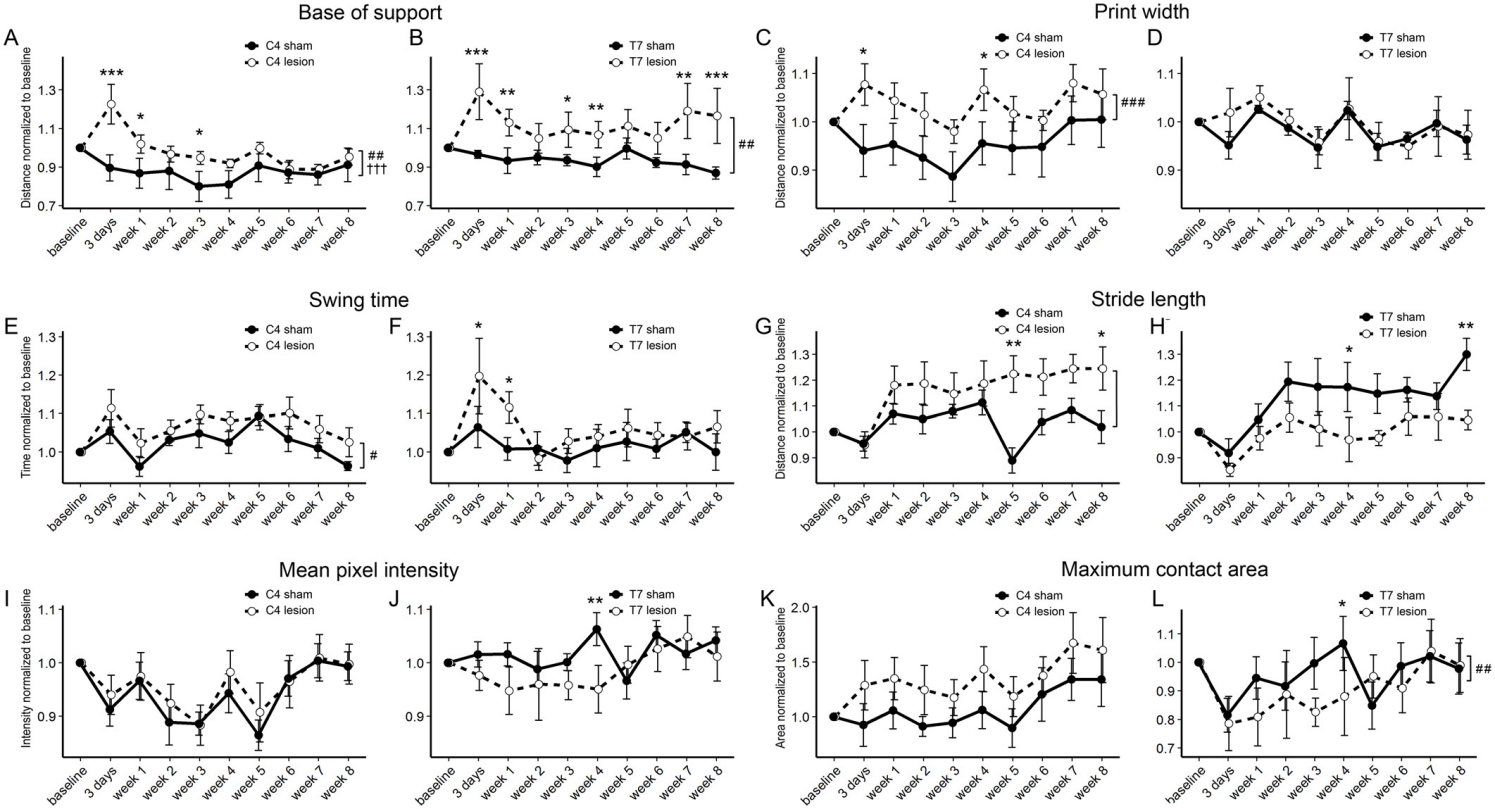
CatWalk gait analysis. Several gait parameters were assessed for eight weeks after cervical or thoracic DC lesion. **(A, B)** Base of support was increased in C4 lesioned animals but slowly returns to sham levels **(A)**, while in T7 lesioned animals the increase is sustained **(B). (C, D)** Hind paw print width was increased in C4 lesioned animals **(C)** but not in T7 lesioned animals **(D)**. **(E, F)** A minor sustained increase in swing time of the hind paws was seen in C4 lesioned animals **(E)** but in T7 lesioned animals a more marked but transient increase occurred **(F). (G, H)** Stride length increased over the time course in C4 injured animals compared to shams while in T7 injured animals stride length was actually decreased at several time points. **(I, J)** Mean pixel intensity was not different in C4 lesioned animals **(I)** and largely unchanged in T7 lesioned animals **(J)**. **(K, L)** Maximum contact area of the hind paws was unchanged after C4 lesion but decreased overall after T7 lesion.**(L)**. Whole time -course comparisons: # p < 0.05, ## p < 0.01, ### p< 0.001 for LESION; ††† p< 0.001 for INT. *p < 0.05, ** p < 0.01, *** p< 0.001 for single time-point comparisons. Error bars are SEM.

Gait parameters were quantified automatically using the CatWalk XT system. The following parameters were analysed for the hind-paws: base of support, stride length, swing time, print width, mean pixel intensity and maximum contact area. In C4 lesioned (Fig 7A) and T7 lesioned (Fig 7B) animals we observed an overall increase in the hind-paw base of support (linear mixed model, p = 0.0002 for C4 INT, p = 0.001 for C4 LESION; p = 0.01 for T7 LESION, T7 INT was n.s.), indicating a widening of the walking pattern of the hind paws in animals with a lesion. Comparing individual time-points, this effect appeared to be more durable in thoracic lesions as significant differences were seen until 8 weeks. In cervical lesions, significant differences were found at early time-points (3 days, 1 week, 3 weeks).

Hind paw print widths of C4 injured animals were overall significantly increased (linear mixed model, C4 INT was n.s., p = 0.0003 for C4 LESION), while no significant differences were observed for T7 injured animals (Fig 7C and 7D).

There was an overall significant increase (linear mixed model, p = 0.01 for C4 LESION, C4 INT was n.s.) in swing time for the hind paws of C4 animals (Fig 7E), which was not observed in T7 injured animals (Fig 7F). We also noted that the hind-leg swing movement in lesioned animals was somewhat exaggerated and circular, consistent with this increase. This deficit appeared to be consistent over the period of eight weeks.

Neither INT nor LESION were significantly different from zero for the stride length parameter after either C4 or T7 lesion (Fig 7G). Stride length in sham animals appeared to increase over time in T7 lesioned but not C4 lesioned animals, and in fact there was a significant interaction of lesion level with time among the sham animals (p = 0.005, by LMM).

No differences were found in the mean pixel intensity of the hind paws in both C4 and T7 lesioned animals (Fig 7I and 7J), while the maximum contact area of the hind paws was significantly decreased in T7 injured (linear mixed model, p = 0.009 for T7 LESION, T7 INT was n.s.), but not C4 injured animals (Fig 7K and 7L). Parameter p-values, F statistics and degrees of freedom are summarized in Table 1.

### Inclined rolling ladder test

Functional deficits after dorsal column injury were also measured in the newly developed inclined rolling ladder test. We found significant deficits in two related binary outcome measures: steps classified as successful steps on smooth bars or any other type (we refer this measure to as Good Smooth Steps), and steps on smooth bars classified as slips or successful (referred to as Slips).

**Fig 8:**
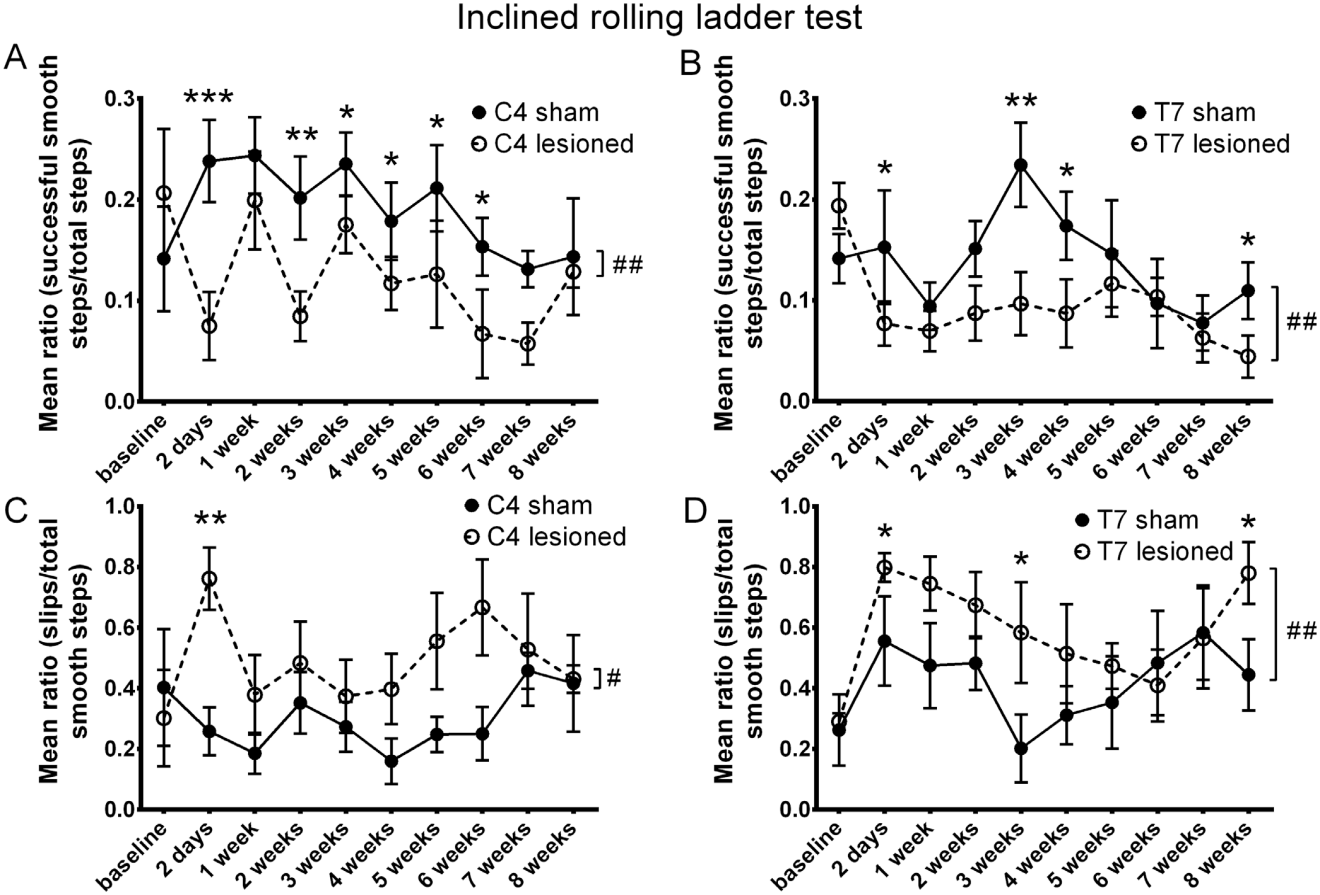
Inclined rolling ladder test. Animals were tested on the inclined rolling ladder test to assess dysfunction related to proprioception and texture perception after cervical or thoracic dorsal column injury. **(A, B)** An overall significant decrease in the Good Smooth Steps measure (successfully executed steps off smooth rungs compared to total steps) was observed for animals that received a C4 **(A)** or T7 **(B)** dorsal column transection. **(C, D)** A significant increase in the Slips measure (slips compared to successful steps off smooth rungs) was seen in both C4 **(C)** and T7 **(D)** injured animals. Whole time -course comparisons: # p < 0.05, ## p < 0.01 for LESION. *p < 0.05, ** p < 0.01, *** p< 0.001 for single time-point comparisons. Error bars are SEM.

We observed an overall significant decrease in the Good Smooth Steps measure in both C4 (Fig 8A) and T7 (Fig 8B) lesioned animals compared to controls (binomial GLMM, p = 0.002 for C4 LESION; p = 0.002 for T7 LESION, C4/T7 INT were n.s.). The Slips measure was also significantly increased in both C4 (Fig 8C) and T7 (Fig 8D) injured animals (binomial GLMM, p = 0.01 for C4 LESION, C4 INT was n.s.; p = 0.001 for T7 LESION, T7 INT was n.s.). These deficits were detectable for six weeks in animals that received a cervical DC transection injury. In both measures the deficits were clearer and more consistent in the cervically lesioned animals. Parameter p-values are summarized in Table 2.

### Comparing tests

**Fig 9:**
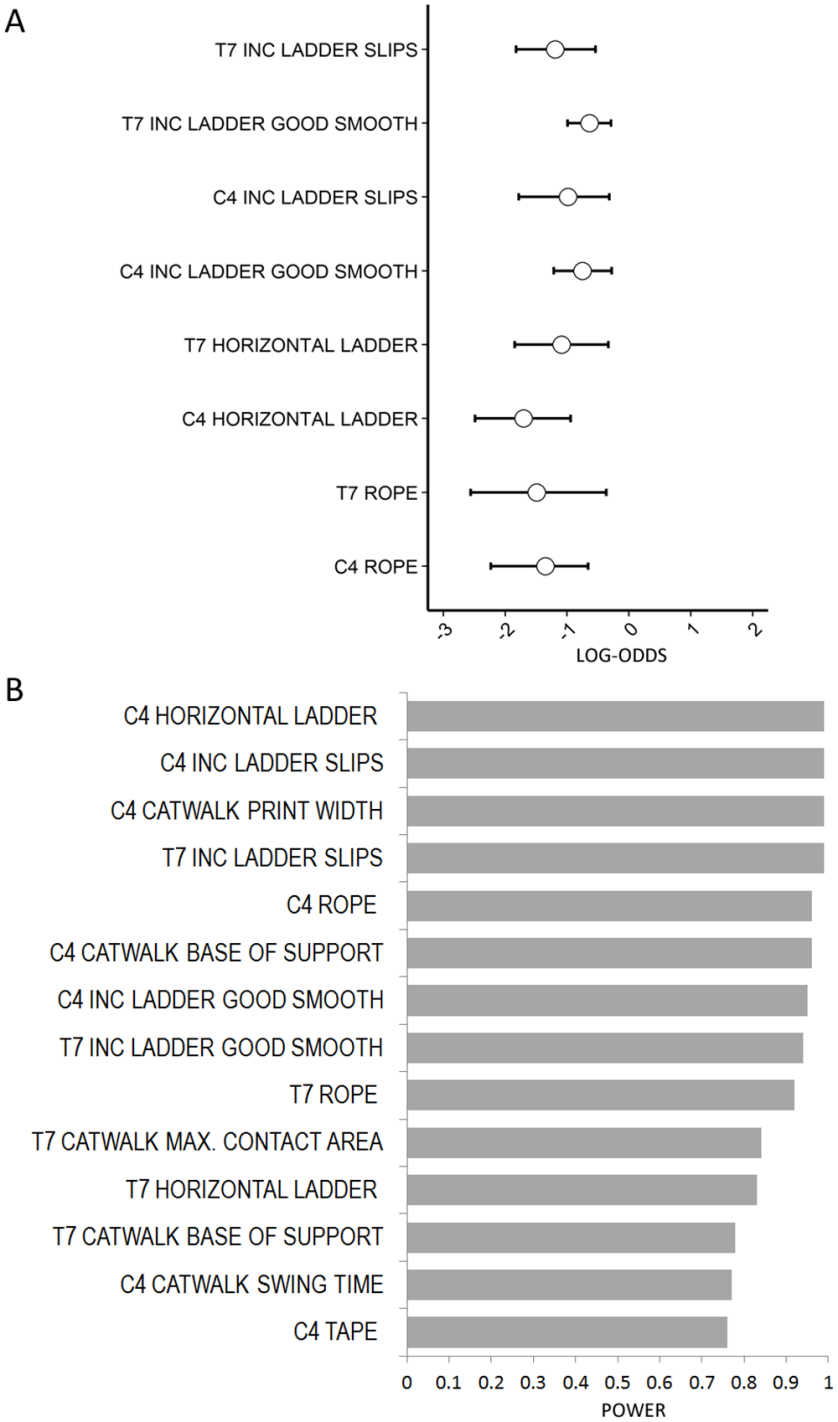
Comparisons of diferent tests and lesion levels. (A) Parameter estimates and 95% confidence intervals for the LESION parameter estimated by GLMM, for C4 and T7 lesions in the rope, horizontal ladder and inclined rolling ladder, on the log-odds scale (x-axis). The effect size and variability may be directly compared for these tests. (B) Power analysis for detection of the effect sizes in LESION seen in this study using n = 6, for all tests at C4 and T7 for which a significant effect was found. Power is shown for α = 0.05.

Because several of these tests use binomial outcomes it is possible to directly compare the effect sizes and uncertainty therein. In Fig 9A parameter estimates and confidence intervals have been plotted for LESION for the rope, horizontal ladder, and both outcomes of the inclined ladder on a log-odds scale. This can be interpreted as the relative effect size of lesioning on test outcome.

To formally compare all the tests and the two lesion levels, we used the data collected here to perform power analyses, to determine the statistical power of each test to detect a difference due to lesioning, using n = 6 with α = 0.05 and with effect sizes that were found here. This was performed for the LESION parameter, only on the tests for which we had found this parameter to be significantly different to zero. The results are presented in Fig 9B. Notably, for all tests the statistical power was equal or greater for C4 lesions compared to T7 lesions, and indeed the majority of the more powerful tests are due to C4 lesions, with the four equally most powerful test/lesion combinations being C4 with horizontal ladder, C4 with inclined ladder (Slips), C4 CatWalk/print width and T7 inclined ladder (Slips).

## Discussion

In this study we have compared several tests to measure recovery of function after cervical and thoracic DC injury in rats. A DC lesion spares other sensory spinal pathways, notably the spinothalamic pathway, so much sensory information still reaches the brain. For this reason it is desirable to use tests which target the modalities carried in the ascending DC. Although a complete DC lesion performed as we describe also unavoidably severs the dorsal corticospinal tract, which may result in minor motor deficits, in this study we focused on tests targeting deficits in proprioception (horizontal ladder, CatWalk gait analysis, rope crossing test) and/or tactile sensation (adhesive tape removal test) and developed the inclined rolling ladder test to assess loss of proprioception and tactile discrimination simultaneously. In addition, for tests where steps can be scored as success or error, we have applied statistical methods that are appropriate for binomially distributed data.

Whole-time course comparisons using linear mixed models and binomial GLMMs proved to be quite sensitive at detecting functional deficits, and in the majority of tests, lesions produced significant deficits over the time -course as a whole. Such binomial tests may well be more powerful than the traditional repeated measures ANOVA, and in any case should be preferred because inferences from tests that assume continuous normally distributed variables are likely to be unreliable when applied to binomial data. At the same time in many tests it was difficult to detect differences at individual time points. Note also that we have not applied multiple testing corrections for these tests and the results should be interpreted in this light. Larger group sizes may allow one to reliably detect differences at later time points where subtle deficits do appear to persist, e.g. in the inclined rolling ladder.

Typically, large deficits were visible at relatively early times but these quickly diminished. It is well known that rodents show considerable adaptive recovery powers following spinal cord injury, and this is in part due to plasticity and remodelling that takes place in the spinal cord after lesion [31–34]. Comparing C4 and T7 lesion levels, both of these should block sensory information from the hind limbs to the brainstem in the dorsal column equally well and the corticospinal tract innervation of the lumbar spinal cord should also be similarly compromised. However, the power analysis performed for the LESION parameter for the various tests at the two different lesion levels indicates that for all tests, equal or greater statistical power was available using C4 lesions. Although one CatWalk parameter (namely hind-paw base of support) showed longer lasting deficits after thoracic lesion, it appears that a cervical lesion is preferable for experimental studies.

We present here a new method to assess recovery of sensory function after DC transection injury. The inclined rolling ladder combines testing of both proprioception and tactile discrimination and with this test we were able to show a consistent deficiency over a period of six weeks compared to sham controls. This indicates that the inclined rolling ladder test shows potential for experiments with treatments aimed at promoting repair of the ascending dorsal columns. Of the two outcome measures tested, the Slips measure had a greater effect size, albeit with greater variation (see Fig 9A) but also had greater power overall. The Good Smooth Steps measure (successful steps on smooth bars versus any other steps), on the other hand, demonstrated statistically significant deficits at more individual timepoints, seen until 6 weeks, and was therefore the measure with the more robust and long-lasting deficits. These outcomes did show variability from week to week, which was probably dependent on the randomised arrangement of bars each week, which will result in varying difficulty for the animals to ascend the ladder. The sensitivity of this test may also be improved by making the ladder longer or increasing the number of test runs performed, to increase the amount of data collected.

The adhesive tape removal test is based on the assumption that impairment of touch sensory input will lead to an increase in the time to notice the sticky tape on the hind paw. We observed an overall deficiency but this was not consistent over time. This test has been used successfully to measure impairment of touch after DC crush lesion [10]. The tape test was also used for assessment of fore limb function after dorsal hemisection and similarly to our findings, an initial large deficiency was seen that returned to control levels at three weeks post injury [35]. The type of tape used in this test could lead to differences in sensitivity. We used a ridged paper type of tape which was described to be effective for testing the front paws of rats [36]. Others used sewed adhesives for bandages and state that these are the best [37]. In our experience the ridged paper adhesive tape was superior to several other smooth paper or synthetic materials, in terms of adhesiveness, stiffness and minimal odor.

The rope crossing test and the horizontal ladder were included based on the hypothesis that deficits in proprioceptive feedback would lead to more stepping errors. For both tests we indeed observed a significant increase in the error rates, which remained until 5 weeks for the horizontal ladder. However, in the rope test the effects disappeared shortly after the initial period. Consistent functional deficits were measured in animals with a DC lesion with a similar horizontal ladder test for up to six weeks [10]. Our data are in agreement with this, and although after 2 weeks the deficit is small, the horizontal ladder test combined with C4 lesions was one of the tests with most overall statistical power. Used with T7 lesions it performed relatively poorly, probably due to a large effect of sham lesioning.

We tested several parameters in the CatWalk gait analysis. In our lesion models, we found increases in hind-paw base of support. This is consistent with the increase in base of support and stride width at 6 weeks after DC crush injury found in [10]. In their footprint analysis stride length was also decreased which we were unable to reproduce. This may be due to a difference in lesion severity, as we performed DC transection lesions, while these authors performed DC crush lesions which may have resulted in a more severe deficiency in sensorimotor function.

Furthermore, in cervical lesions, we found that hind paw swing time appears to be slightly increased for the entire 8 week period, although this deficit was only significant over the time-course as a whole. However with greater statistical power this outcome may be useful. This deficit may directly reflect lack of proprioceptive function as it appears to be related to exaggerated circular hind limb stepping movements ('windmilling').

We also assessed the hind paw parameters 'mean pixel intensity' and 'maximum contact area' intended to detect changes in weight bearing and paw placement. The 'mean pixel intensity' parameter has been used by others to assess mechanical allodynia in neuropathic pain models, however we observed no change in this parameter after thoracic or cervical DC transection injury [38–40]. The 'maximum contact area' parameter did respond significantly to thoracic, but not cervical, lesions, in contrast to the other tests.

Our results contrast with those of [41] who found that after a lesion of the DC by aspiration no differences were found between injured and non-injured animals on a gridwalk test and in base of support, stride length and rotation angle in a platform locomotion task, although consistent deficits in these tests were found after a dorsal hemisection.

In a number of studies in which a cervical DC transection lesion was performed, several deficits in forepaw, but not hindpaw function, were found [11,12,42]. No deficit was found of the hind paws on a horizontal ladder test or in stance duration, stride length and stride duration on a platform locomotor task during eight weeks after a cervical DC transection or a deeper DC lesion that also fully transected the corticospinal tract [11]. Consistent with our results, in a comparison of cervical and thoracic DC transections a cervical DC injury led to larger deficits than thoracic injury [12]. Both cervical and thoracic lesions resulted in transient deficits in a horizontal ladder test that was performed at two time points (at 2 weeks and 6 weeks after injury). Again, stance duration, stride length and stride duration on the platform locomotor task were unaffected after both levels of injury. These studies and our results indicate that transection lesions of the DC lead to mild and transient deficits in hind paw function.

In summary, we show here that although a number of classical functional tests can be used to measure an overall decline in sensorimotor function after DC transection injury, for many tests spontaneous recovery occurs within several weeks, such that functional deficits become too small to detect at individual time points. This is particularly true of the tape removal task and the rope test, tests which also suffered from high variability. However, whole time-course comparisons are more sensitive and on many tests deficits are readily detectable, with the limitation that this does not give any information about when these deficits are prominent. Nonetheless, the dorsal column injury model allows a limited time window for experimental treatment induced functional recovery to be detected. The horizontal ladder and the inclined rolling ladder which we have presented here appear to be the most sensitive overall. The use of binomial GLMMs with success/error scoring schemes is appropriate with such tests. Cervical lesions appear to produce more easily detectable and longer lasting deficits than thoracic lesions on these two tests, with deficits continuing for at least 5–6 weeks. Some Catwalk parameters are also sensitive to the lesion, particularly the hind-paw base of support and hind-paw print width. The horizontal ladder, inclined rolling ladder and the Catwalk may prove useful for measuring functional recovery following experimental intervention in dorsal column injury models.

## Abbreviations

CTB: cholera toxin subunit B
DC: dorsal columns
GLMM: generalized linear mixed model
LMM: linear mixed model
